# *In Vitro* Functional Analyses of Infrequent Nucleotide Variants in the Lactase Enhancer Reveal Different Molecular Routes to Increased Lactase Promoter Activity and Lactase Persistence

**DOI:** 10.1111/ahg.12167

**Published:** 2016-10-07

**Authors:** Anke Liebert, Bryony L. Jones, Erik Thomas Danielsen, Anders Krüger Olsen, Dallas M. Swallow, Jesper T. Troelsen

**Affiliations:** ^1^ Research Department of Genetics Evolution and EnvironmentUniversity College LondonLondonUK; ^2^ Department of Science and EnvironmentRoskilde UniversityRoskildeDenmark

**Keywords:** Lactase persistence, enhancer, transfection, gel shift, supershift, transcription factor

## Abstract

The genetic trait that allows intestinal lactase to persist into adulthood in some 35% of humans worldwide operates at the level of transcription, the effect being caused by *cis*‐acting nucleotide changes upstream of the lactase gene (*LCT*). A single nucleotide substitution, ‐13910 C>T, the first causal variant to be identified, accounts for lactase persistence over most of Europe. Located in a region shown to have enhancer function *in vitro*, it causes increased activity of the *LCT* promoter in Caco‐2 cells, and altered transcription factor binding. Three other variants in close proximity, ‐13907 C>G, ‐13915 T>C and ‐14010 G>C, were later shown to behave in a similar manner. Here, we study four further candidate functional variants. Two, ‐14009 T>G and ‐14011 C>T, adjacent to the well‐studied ‐14010 G>C variant, also have a clear effect on promoter activity upregulation as assessed by transfection assays, but notably are involved in different molecular interactions. The results for the two other variants (‐14028 T>C, ‐13779 G>C) were suggestive of function, ‐14028*C showing a clear change in transcription factor binding, but no obvious effect in transfections, while ‐13779*G showed greater effect in transfections but less on transcription factor binding. Each of the four variants arose on independent haplotypic backgrounds with different geographic distribution.

## Introduction

The expression of the intestinal enzyme lactase, which is critical for the majority of Eutherian mammals during the suckling period, is generally downregulated after weaning, but persists into adult life in some humans. This trait, known as lactase persistence (LP, MIM 223100), is carried by some 35% of the world's population and is notably more frequent in populations with a long history of pastoralism and dairy farming (Ingram et al., 2009a).

It has been shown that the differential expression of human lactase (lactase–phlorizin hydrolase, LPH, EC 3.2.1.108) in adults is regulated at the level of transcription and the effect is *cis*‐acting to the lactase gene (*LCT*) (Ho et al., 1982; Flatz, 1984; Wang et al., 1995; Kuokkanen et al., 2003. A single nucleotide substitution, ‐13910*T (rs4988235), about 14 kb upstream of the *LCT* transcriptional start site, in intron 13 of the neighbouring Minichromosome Maintenance 6 (*MCM6*) gene was the first causal variant to be identified (Enattah et al., 2002) and accounts for LP over most of Europe (Enattah et al., 2002; Kuokkanen et al., 2003; Poulter et al., 2003; Enattah et al., 2007). It is located in a region shown to have enhancer function *in vitro* (Olds & Sibley, 2003; Troelsen et al., 2003; Lewinsky et al., 2005) and compared to the ancestral variant, the presence of ‐13910*T results in about twofold increased enhancer activation of the *LCT* promoter in human intestinal–derived Caco‐2 cells, as well as altered transcription factor binding (Troelsen et al., 2003; Lewinsky et al., 2005; Ingram et al., 2007; Olds et al., 2011), indicating that this variant accounts for LP in its carriers, and this functional effect is supported by *in vivo* studies in mice (Fang et al., 2012).

During the course of screening the *LCT* enhancer sequence for novel variation, we and other investigators found a number of other variants clustering in close proximity to each other. In the first studies, three further functional alleles were identified in Middle Eastern and African populations: ‐13915*G (rs41380347), ‐13907*G (rs41525747) and ‐14010*C (rs145946881). These also showed increase reporter gene expression compared to the ancestral variants and/or differences in protein‐DNA binding affinity (Ingram et al., 2007; Tishkoff et al., 2007; Enattah et al., 2008, Jensen et al., 2011, Olds et al., 2011).

Several other variants have been found in the enhancer region (e.g. Ingram, 2008; Ingram et al., 2009b; Jones et al., 2013; Baffour‐Awuah et al., 2015). Some have been shown not to be positively associated with lactose digester status [e.g. ‐13913 T>C (Jones et al., 2013)], while others were candidate LP variants. Four of these allelic variants were chosen for *in vitro* functional tests and the results are reported here.

‐14009*G (rs869051967) was selected because it was strongly associated with digester status (Jones et al., 2013), and ‐14011*T (rs4988233) (Lember et al., 2006; Friedrich et al., 2012; Gallego Romero et al., 2012; Jones et al., 2013), although too rare to test for association, is also located immediately adjacent to the known functional variant ‐14010*C. ‐13779*G (rs527991977) was of interest because it was found to be relatively common in some groups in India, in the milk drinking Toda, for example, though it was also found in hunter‐gatherers (Gallego Romero et al., 2012). ‐14028*C (rs759157971) had previously been found as the only *LCT* enhancer allele identified in the second highest expressing transcript of a homozygous lactase persistent person (Poulter et al., 2003; Ingram, 2008). Here, we report functional analysis for these four derived variant alleles in comparison with the ancestral sequence using cell culture transfection and gel shift experiments. We tested both undifferentiated and differentiated Caco‐2 cells in this study, since it is well known that intestinal hydrolyses increase in their expression in the course of differentiation of this cell line, and that there are alterations in expression of the associated transcription factors (Chantret et al., 1988; Boyd et al., 2010). Preliminary results for the effect of ‐14009*G in transfection experiments have been reported recently by our group (Jones et al., 2013) but here the function of this site is investigated in much more detail.

We show that the three adjacent variant alleles ‐14009*G, ‐14010*C and ‐14011*T have similar and clear effects on upregulation of promoter function, as assessed by transfection, but are each involved in different molecular interactions. The results obtained for ‐13779*G and ‐14028*C were also suggestive of function but were less conclusive.

## Materials and Methods

### Cell Culture

Human Caco‐2 cells were cultured in Dulbecco's modified Eagle's medium, supplemented by 100 U/ml penicillin, 100 μg/ml streptomycin and 10% foetal calf serum. Cells were kept in a humid environment at 37°C and 5% CO_2_ and split at 80% confluence (after 3–4 days), and left to differentiate for 13 days after seeding, before preparation of nuclear extracts, as described elsewhere (Ausubel et al., 2002; Troelsen et al., 2003).

For gel shift assays, sense and antisense oligonucleotides (Table S1) were annealed and radioactively 5′ labelled using T4 Polynucleotide Kinase (Fermentas, Thermo Fisher, Copenhagen, Denmark) and 25–35 μCi [γ^32^P]ATP (Perkin Elmer, Skovlunde, Denmark). The probes were purified using MicroSpin G‐25 Columns (Illustra, GE Healthcare, Brondby, Denmark).

Each protein/DNA binding reaction contained 4.5–9.0 μg differentiated Caco‐2 nuclear extract, 4 μl dialysis buffer (20 mM HEPES [pH 7.9], 20% glycerol, 1.5 mM MgCl_2_, 100 mM KCl, 0.2 mM EDTA, 0.5 mM DTT) and 10 μl gel shift buffer (25 mM Tris‐HCl [pH 7.8], 5 mM MgCl_2_, 6 mM KCl, 0.5 mM EDTA, 1 mM DTT, 1 μl/ml protease inhibitor cocktail [Sigma‐Aldrich, Brondby, Denmark], 5% Ficoll [PM 400], 2.5% glycerol). A total of 0.25 μg poly‐dI‐dC (Sigma‐Aldrich, Brondby, Denmark) and 2.5 pmol of unlabelled unspecific oligonucleotides were added to minimize nonspecific DNA binding, though some nonspecific binding is unavoidable. Bands were classified as specific when they were consistently present in several experiments and were reduced or removed when competed with specific unlabelled oligonucleotides and not with unspecific unlabelled oligonucleotides.

To identify proteins forming the DNA–protein complexes, specific antibodies were used to obtain evidence of change in electrophoretic pattern (decrease in electrophoretic mobility, known as supershift, or reduction of band intensity). A total of 1–2 μl of antibodies to Cdx‐2 (BioGenex, Brondby, Denmark), Oct‐1, (Hepatic Nuclear Factor) HNF‐1α, HNF‐4α, Ets‐1/2 or Ets‐1 (all Santa Cruz Biotechnology, Aarhus, Denmark) were used. As a second approach competition assays were carried out, for which 2.5 pmol of the unlabelled annealed competitor oligonucleotide was added to the reaction. After incubation of the mixture for 10 min on ice, 2.5 fmol of probe was added and another incubation step followed for 20 min on ice. The DNA–protein complexes were subjected to electrophoresis on 5% nondenaturing polyacrylamide gels, which were analysed using phosphorimaging instrument (Molecular dynamics, GE Healthcare, Brondby, Denmark).

### Cell Culture Transfections (Luciferase Reporter Studies)

The *LCT* enhancer variants were generated by site‐directed mutagenesis in a two‐step Polymerase Chain Reaction (PCR) amplification of the pGL3 hLPH1085‐13910C enhancer plasmid construct (Troelsen et al., 2003). First, the region was amplified in two separate PCRs to generate a left fragment containing a 5′ *Bam*HI and a right fragment containing a 3′ *Sal*I restriction site. The two fragments were then annealed to create an overlap at the mutated position and amplified in a second PCR to create the whole 450 bp fragment, containing the restriction sites at both ends.

All enhancer fragments were TA‐cloned into the pCR 2.1‐TOPO plasmid (Invitrogen) and later inserted into pGL3 hLPH1085 with *Sal*I and *Bam*HI digest. The plasmids are shown in Figure S1A. The sequences of all fragments were verified by sequencing and the “ancestral” sequence is shown in Figure S1B.

Caco‐2 cells were grown to 80% confluence and plated in 24‐well plates the day before transient transfection, with each well containing approximately 5 × 10^4^ Caco‐2 cells. Each transfection experiment was carried out as four or eight repeats. Cells were transfected with a total DNA amount of 0.3 μg per well including 0.05 μg luciferase reporter gene plasmid, 0.025 μg pCMV‐lacZ plasmid (Promega) and 0.225 μg pBluescript SK^+^ plasmid (Stratagene) in a 25 μl volume containing 15 mM NaCl. A total of 25 μl of transfection reagent containing 2 μM polyethyleneimine (PEI) in form of Exgen 500 (22 kDa, Fermentas) in 10 mM NaCl solution or PEI25 (25 kDa, Alfa Aesar) were added to the DNA mix.

After 2 and 9 days of transfection, the Caco‐2 cells were harvested and luciferase and β‐galactosidase activity were measured using the Dual‐Light chemiluminescent reporter gene assay (ABI) (Martin et al., 1996; Bronstein et al., 1997). Luciferase activity was normalized against β‐galactosidase activity by calculating relative luciferase/β‐galactosidase ratios for each well, and difference between the transfection results tested for statistical significance with a Student's unpaired *t*‐test. Consistency of effect of the different variants in four independent experiments was tested by a two‐way (Analysis of Variance) ANOVA (using GraphPad Prism software, version 6.0d).

### Haplotype Background of Variants

Eight of the 11 core haplotype markers, previously defined by Hollox et al. (2001) were used to determine the most likely *LCT* haplotype background: ‐958 C>T (rs 56064699), ‐946 A>G, ‐943/42 TC>ΔΔ (rs148142676), ‐942 C>G, ‐875 G>A (rs78205226), 678 A>G (rs562211644), 666 G>A (rs3754698), 5579 T>C (rs2278544). The (Simple Nucleotide Polymorphism) SNPs 666 G>A, 5579 T>C were genotyped using the KASP system (LGC Genomics). Typing of the other haplotype defining markers was done by Sanger sequencing, as was the *LCT* enhancer region. Haplotypes were inferred using PHASE [version 2.1.1 (Stephens et al., 2001; Stephens & Donnelly, 2003; Stephens & Scheet, 2005)] and results were inspected by eye.

### Bioinformatic Analysis

Sequences for transcription factor binding sites were extracted from binding motives stored in the TRANSFAC database [TRANSFAC Professional version 12.1, BIOBASE Biological Databases (Matys et al., 2006)]. To search for possible transcription factors binding to the variant oligonucleotides, the MATCH tool (Kel et al., 2003) of the TRANSFAC database was used.

### Human Samples

DNA samples used in this study were collected anonymously with fully informed consent of the donors, with permissions obtained from the relevant local authorities, under ethical approvals for population genetics and LP (ULCH 99/0196, 01/0236 and UCL 2670/001).

## Results

### Transfections

Luciferase reporter gene expression of the different enhancer variant constructs was measured in undifferentiated (2 days after transfection) and differentiated Caco‐2 cells [9 days after transfection, signs of differentiation visible as described by Pinto et al. (1983)]. Constructs containing the LP alleles ‐13910*T and ‐14010*C were included in the experiments as controls since they were known to influence reporter gene expression, at least in undifferentiated cells (Troelsen et al., 2003; Tishkoff et al., 2007; Jensen et al., 2011).

**Figure 1:**
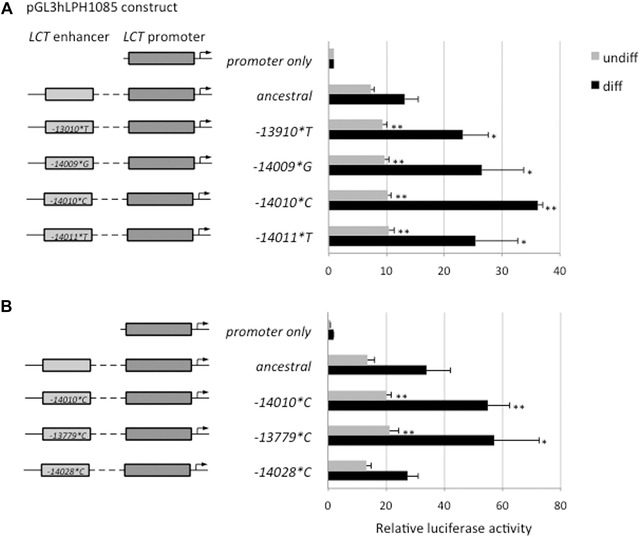
Representative experiments showing luciferase reporter assays for *LCT* enhancer variants in undifferentiated (undiff) and differentiated (diff) Caco‐2 cells (after 2 and 9 days of transfection, respectively). Luciferase activity of enhancer variant constructs are shown relative to the promoter only construct: (A) the new variant alleles ‐14009*G and ‐14011*T located adjacent to ‐14010*C, (B) the other two variants tested, ‐13379*C and ‐14028*C. Luciferase activities (means ± SD, *n* = 4) were corrected for transfection efficiency against ß‐galactosidase and normalized to the expression of pGL3 hLPH1085 (promoter only). Significant differences of luciferase activity compared to the ancestral variant were calculated using a student's *t*‐test and are shown by ** (*P* < 0.01) and * (*P* < 0.05). Maps and sequences of the plasmids are shown in Figures S1A and B.

The constructs containing the alleles ‐14009*G and ‐14011*T show increased luciferase expression in both undifferentiated and differentiated Caco‐2 cells compared to the ancestral construct that carries T at ‐14009 and C at ‐14011 (Fig. 1). Over four independent experiments, each consisting of four (or for differentiated cells in one case eight) replicates, the average expression was increased approximately 1.4‐ and 1.5‐fold in undifferentiated and 1.9‐ and 1.8‐fold in differentiated cells for ‐14009*G and ‐14011*C, respectively compared with the ancestral sequence (see Figure S3). The ‐13779*C construct also showed a consistent increase in reporter gene expression in both undifferentiated and differentiated cells and, despite a high intraexperimental variance, was highly significant across all experiments, as tested by two‐way ANOVA. In contrast to the other variant, alleles ‐14028*C did not show any evidence of influence on enhancer activity.

#### Electrophoretic mobility shift essays (EMSAs)

To further investigate the influence of the four new variant alleles on the *LCT* enhancer, electrophoretic mobility shift essays (EMSAs) were performed to evaluate differences in DNA binding properties to nuclear proteins extracted from Caco‐2 cells.

−14009T>G,−14010G>C and 14011C>T

**Figure 2:**
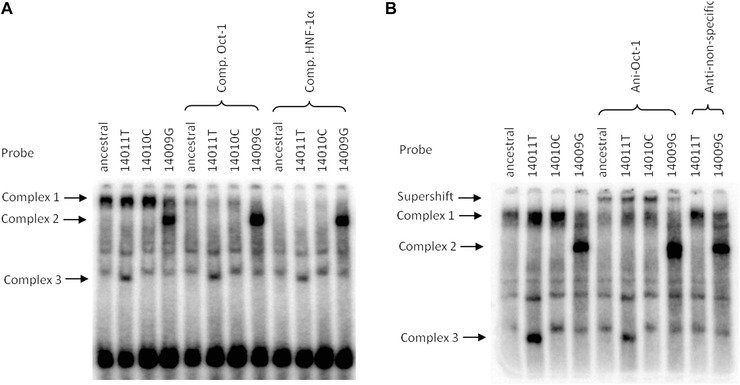
Phosphorimages of gel shift assays of competition (A) and supershift experiments (B) for the ‐14011T, ‐14010C and ‐14009G variant probes compared to the ancestral version. Competitors (Comp.) covering known binding sequences for TFs and antibodies (Anti‐) were used as indicated above the gel images, a nonspecific antibody was used as negative control. Different probe–protein complexes were formed of which the upper ones (Complex 1) could be competed with Oct‐1 and HNF‐1α competitors (A) and supershifted (B) with an Oct‐1 antibody for all four probes, whereas the DNA–protein complex formed with the ‐14009G probe (Complex 2) was not shifted. A slightly different binding pattern was seen for the lower band for ‐14011T (Complex 3). Note that the ancestral version of the sequence used in these experiments is ‐14009T, ‐14010G, ‐14011C (see Table S1).

To examine the effect of the ‐14009 T>G and ‐14011 C>T substitutions flanking the previously reported ‐14010 G>C functional variant, oligonucleotides were designed to cover these positions, and were derived from the same length of sequence used previously to study ‐14010 G>C (Jensen et al., 2011). A ‐14010C variant probe was thus also tested for comparison in all experiments, as well as the probe with the ancestral nucleotides at each position. Gel shift analysis revealed similarities of all three derived probes but also major differences (Fig. 2).

In each case, the gel shifts show a low mobility band (Complex 1), which is more intense for the ‐14011T and ‐14010C variant probes than for ‐14009G or the ancestral probe, as observed previously for ‐14010C. The sequence overlaps an Oct‐1 and HNF‐1α binding site (Jensen et al., 2011) and the shifted band was reduced by both the HNF‐1α and the Oct‐1 competitor, with slightly stronger competition with HNF‐1α (Fig. 2A). Binding of Oct‐1 was confirmed with an Oct‐1 antibody resulting in a “super‐shifted” protein–DNA complex, generated with all variant probes (Fig. 2B), and no shift but a slight inhibition of the complex formation was seen with the HNF‐1α antibody (not shown).

In the case of ‐14011T, there was also a slightly different higher mobility DNA‐protein complex (Complex 3). Binding of GATA‐3 and 4 specifically to ‐14011T was predicted bioinformatically. However, preliminary experimental results with different GATA competitor oligonucleotides point to a competition of both the ancestral as well as the derived ‐14011 probes (data not shown).

The ‐14009G probe showed a pattern that differed more dramatically from the three other probes tested at that locus, in that a prominent additional DNA–protein complex was formed (Complex 2 in Fig. 2), which was not affected by the Oct‐1 and HNF‐1α competitors or antibodies. This suggests that another protein in the intestinal nuclear extract is specifically binding to the ‐14009G containing sequence.

**Figure 3:**
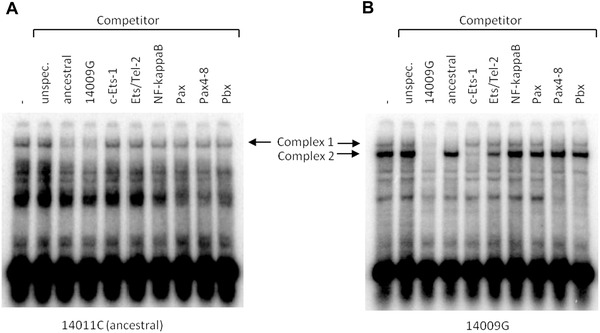
Phosphorimages of EMSAs of comparing the ancestral (A) and derived (B) variant probes of the *‐14009T>C* SNP. Competitors were used as indicated above the images. The specific DNA–protein complex for ‐14009G (Complex 2) could not be competed with the corresponding ancestral sequence competitor (Table S1) but was with competitor oligonucleotides of the Ets family.

Bioinformatic analyses predicted additional transcription factors that might potentially bind to the derived sequence, with G at position ‐14009, but not to the ancestral sequence. These were members of the Ets transcription factor family, as well as others for which the difference was less marked (NF‐kappaB, Pax, Pax4‐8 and Pbx). Competitor oligonucleotides were designed taking into account specific transcription factor binding motifs found in TRANSFAC, or using information about matching competitors available from the Affymetrix EMSA kit (Table S1) and Figure 3 shows the EMSA experiments with some of these competitors, comparing the ancestral sequence and ‐14009G. The upper DNA–protein complex formed with both the ‐14009*G* and the ancestral probes (Complex 1) showed a very slight competition of the binding for NF‐kappaB, Pax, Pax4‐8 and Pbx. However, ‐14009G complex 1 could be fully competed with the ancestral sequence. The prominent lower band (Complex 2 in Fig. 3), obtained with the ‐14009G probe could not, on the other hand, be competed with ancestral probe, confirming the specificity of the factor binding to the derived allele. All Ets family oligonucleotides, c‐Ets‐1, Aff_ETS (1) and Aff_ELK‐1 competed with the ‐14009G specific band, but the c‐Ets‐1 sequence had the greatest effect on the formation of this complex. Interestingly, the upper band (Complex 1) was also competed with the Aff_ELK‐1 competitor in both the ancestral and the ‐14009G probes (not shown).

The reciprocal experiments, using c‐Ets‐1 and Aff_ELK‐1 as probes showed that the c‐Ets‐1 probe was more effectively competed with ‐14009G than with the ancestral sequence, whereas Aff‐ELK‐1 was competed with both competitor sequences (not shown).

−13779G>C

**Figure 4:**
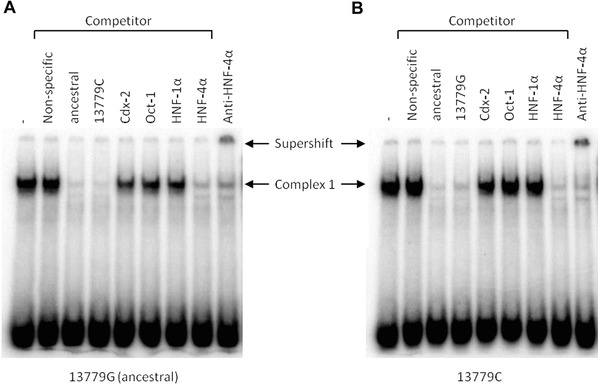
Phosphorimages of EMSAs comparing the ancestral (A) and derived (B) variant probes of the ‐13779 G>C SNP. Competitors with different binding sequences for TFs or an oligonucleotide with a nonspecific sequence as well as an antibody (Anti) against HNF‐4α were used as indicated above the pictures. The specific DNA–protein complex (Complex 1) formed with both probes could clearly be competed with the HNF‐4α competitor and supershifted with the antibody to HNF‐4α.

Further gel shifts, examining the ‐13779 G>C SNP, revealed a strong DNA–protein complex for both the ancestral and derived variant probes that could be competed in a similar way with the unlabelled probe containing either the ancestral or derived allele (Fig. 4). An HNF‐4α binding site was bioinformatically predicted for the derived allele but not the ancestral sequence. However, complete competition of the band was seen for both probes with the oligonucleotide containing an HNF‐4α binding site. This binding could be confirmed with the supershift of the complex formed with the HNF‐4α antibody also with no allele specific difference. Both variant probes showed a slight competition with the Cdx‐2 competitor and only the ancestral allele ‐13779G showed a minor change in band intensity with the Oct‐1 and HNF‐1α competitors (Fig. 4).

−14028T>C

**Figure 5:**
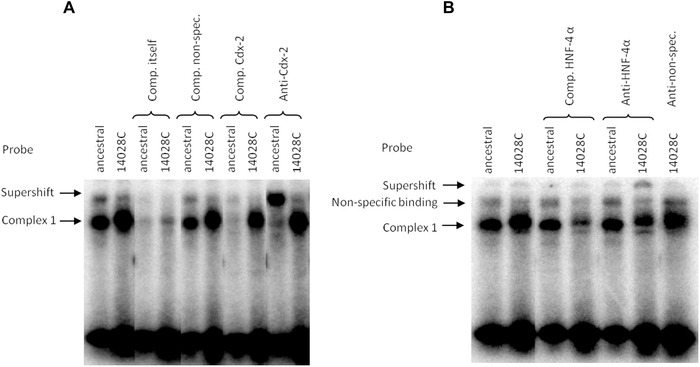
Phosphorimages of EMSAs comparing the ancestral and derived variant of the ‐14028 T>C SNP. Competition experiments (Comp.) with unlabelled variant oligonucleotides for a specific competitor for Cdx‐2 (A) or HNF‐4 α (B) indicate binding specificity of these TFs, which is shown further by supershift experiments with antibodies (Anti) against Cdx‐2 (A) and HNF‐4 α (B). A nonspecific antibody was used as negative control (B). Note that lanes were cut out of gel pictures but all remaining lanes are from the same gels. Bands were classified as nonspecific binding when they were only present on some of the gels and were reduced or removed with competition of nonspecific oligonucleotides.

Probes corresponding to both alleles of the ‐14028T>C SNP bound strongly to proteins of the nuclear extract (Fig. 5). This binding could in both cases be slightly competed with unlabelled oligonucleotides containing Oct‐1 and HNF‐1α binding sites (not shown). However, the ancestral ‐14028T probe was strongly competed with the Cdx‐2 competitor sequence and specific binding of this protein to ‐14028T was confirmed in supershift experiments. Binding to HNF‐4α of the derived ‐14028C probe but not the ancestral one was predicted bioinformatically and indeed, the DNA–protein bands with the ‐14028C probe were decreased with the addition of an HNF‐4α competitor and a supershift with the HNF‐4α antibody confirmed that this transcription factor is binding to the ‐14028C probe (Fig. 5).

### Haplotype Backgrounds

In order to determine whether they were likely to have a common origin, and or whether there were other likely sequence differences located within the region of the constructs, the haplotype background of each of the derived alleles studied here was inferred by genotyping of key markers (Hollox et al., 2001) in as many of the samples as were readily available and inferring the phase of the alleles using PHASE. They each appear to occur on a specific and different haplotype background with the exception of ‐14011*T, which occurred most frequently on A (like ‐13910*T and 13907*G) but twice on B (Table 1).

**Table 1:** Occurrence of rare derived *LCT* enhancer alleles in populations clustered by country, and haplotype background defined as in Hollox et al. (2001)

	Variant					
Geographic region	‐14028 T>C rs759157971	‐14011 C>T rs4988233	‐14010 G>C rs145946881	‐14009 T>G rs869051967	‐13779 G>C rs527991977	*n* chromosomes
Europe/Asia						
Belarus	–	1	–	–	–	100
England	1	–	–	–	–	140
Greece	–	1	–	–	–	120
India	–	–	–	–	3	222
Italy	–	1	–	–	–	258
Mongolia	–	1	–	–	–	166
Ukraine	–	1	–	–	–	74
Russia	–	1	–	–	–	252
Sweden	–	2	–	–	–	130
Middle East						
Kuwait	–	–	–	1	–	66
Iran	–	1	–	–	–	154
Syria	–	–	–	–	1	140
Yemen	–	–	1	–	5	166
Africa						
Ethiopia	–	–	1	7	–	370
Sudan	–	–	–	25	–	204
Tanzania	–	–	12	–	–	82
Haplotype	**B**, G	**A**, J; **B**, G	**P**, W, Y	**X**, m, n	**C**, M, j, k	
(inferred from n chromosomes tested)	2	7; 2	14	33	5	

Sequence and haplotype data include samples of the African populations from Jones et al. (2015) and one previously published additional individual carrying the ‐14028*C allele (Poulter et al., 2003; Ingram, 2008) was used for haplotyping that allele. In the case of ‐13779*C, the samples from Yemen only had complete data for all the haplotype markers. Core *LCT* haplotype in bold is the most likely given the markers tested. Previously published information on the populations carrying ‐13779*C and ‐14011*T in India is shown in Table S2.

## Discussion

In this paper we have found, using the classical approaches of transfection and gel shift assays, new evidence for function *in vitro*, of four relatively infrequent variants located in the *LCT* enhancer.

It had previously been shown that ‐14010 G>C is located in an Oct‐1 binding site (‐14021 to ‐14009 upstream of *LCT*) and resides close to an HNF‐1α binding site (‐14007 to ‐13993; Figure S2) and the C substitution alters gene transcription *in vitro* in Caco‐2 cells (Jensen et al., 2011). Here we study two further candidate functional variants, ‐14009 T>G and ‐14011 C>T, adjacent to the well‐studied ‐14010 G>C variant, and show that they also have a clear effect on promoter activity upregulation as assessed by transfection assays, but notably are involved in different molecular interactions. The results for the two other variants (‐14028 T>C, ‐13779 G>C) were suggestive of function, ‐14028*C showing a clear change in transcription factor binding, but no obvious effect in transfections, while ‐13779*G showed greater effect in transfections but less on transcription factor binding. Each of the four variants arose on independent haplotypic backgrounds and have different geographic distributions.

Our experiments on the three neighbouring variants show that they have rather different effects on protein binding, which nevertheless leads to the same transcriptional outcome as the derived variant ‐14010*C reported earlier. All three variant probes and the corresponding ancestral sequence show evidence of binding to Oct‐1 but this is slightly stronger for the ‐14010C and ‐14011T variant probes. Note that ‐14009G on the other hand showed very clear binding to yet another transcription factor with distinct electrophoretic mobility. The bioinformatic analysis followed by the use of target‐designed competitors shows very good evidence that this is a member of the Ets family, possibly c‐Ets‐1, even though it has not yet been possible to confirm this in supershift experiments using currently available antibodies.

Ets (E26 transformation‐specific) factors are known to interact with other proteins to regulate DNA binding or transcription and to play an important role in the differentiation, survival and proliferation of cells, including those of the intestine (Sharrocks, 2001; Galang et al., 2004; Hsu et al., 2004, Seth & Watson, 2005; Hollenhorst et al., 2011; Findlay et al., 2013).

The variant at ‐14028 T>C is located within a proposed Cdx‐2 binding site (Lewinsky et al., 2005). Here, we confirm this binding site experimentally since a clear supershift could be seen for ‐14028T (ancestral probe) with a Cdx‐2 antibody in EMSAs. We also found *in vitro* evidence for alteration of transcription factor binding at that locus since the C substitution caused a loss of binding to Cdx‐2 and generates a HNF‐4α binding site. In view of the fact that there is such a marked difference in the transcription factor binding, and the strong prior evidence of high allelic expression, (see Figure S4) the lack of effect in transfection experiments, at least under the conditions tested, was surprising.

This led to consideration of the possible significance of allelic differences in the haplotypic background of the reporter gene construct. The construct (Figure S1B) carries the *LCT* promoter region from ‐1097 to ‐13, and there are known polymorphic sites at positions ‐958, ‐946, ‐943/2, ‐875, ‐678 and 559/552 and positions within that sequence (Harvey et al., 1998; Hollox et al., 2001). The promoter used for these constructs is the same throughout all the experiments and carries the derived *T at ‐958 and the ancestral variants at the other positions (see Figure S1). Early experiments showed that ‐958*T disrupts binding to an unknown transcription factor (Hollox et al. 1999), and a decreased expression in transfection experiments has also been reported for ‐958*T (Chitkara et al., 2001), suggesting that this site plays a functional role. Interestingly, this sequence is identical to that of the haplotypic background of the derived ‐14028*C and in fact different from that of the other derived enhancer alleles tested here. Even though this effect is counterintuitive, it seems likely that there are combinatorial effects with SNPs at sites others than in the enhancer. The combined effect of these different variants *in vitro* may well differ from that *in vivo*. Another subtlety is that the ‐14133 to ‐14020 gene‐regulatory region has been shown to decrease enhancer function in reporter gene assays rather than increase it (Jensen et al., 2011).

Although the reporter experiments to study ‐13779 G>C gave high variance for reasons that were not clear, an increase in expression was seen consistently. The probes were shown to contain a HNF‐4α binding site, with no allelic difference but slight difference in Cdx‐2 binding. The suggestive evidence for a functional role of ‐13779*C had been not very strong since it had in fact previously been reported in one Somali individual who was diagnosed as a lactose mal‐digester (Ingram et al., 2009c). But its occurrence, in two South Indian pastoralist groups, the Toda and Yadava, at higher frequencies than most of their nonpastoralist neighbours in Tamil Nadu, initially prompted us to consider it as a potential functional variant, although it was also found in one of the Tamil Nadu Hunter‐Gatherer groups, the Kurumba, at relatively high frequency as well as in one hunter‐gatherer group in Kerala (Table S2, Gallego Romero et al., 2012). Curiously at least one functional allele has also been found in African hunter‐gatherers (Ranciaro et al., 2014) and it is tempting to speculate that these alleles may have originated in hunter‐gatherer with an early selection pressure operating through glycosides that occur in plants, before spread into other groups and subsequent selection for milk drinking. Despite these uncertainties, the combined results suggest that this variant is worth pursuing further as a candidate locus.

The results presented here provide further strong support for the functional role of ‐14009*G and indeed more support for the functional role of this small upstream region, which also houses ‐14010*C. The pattern of distribution of ‐14009*G in East African milk drinkers (Jones et al., 2015) is indicative of selection whereas this is not the case for ‐14011*T. Although ‐14011*T would appear to be functional and most of the alleles found so far are located on the same or similar A haplotype background, they are very rare, and scattered over many countries, including India (Table S2), failing to show any noticeable impact of selection. The two occurrences of ‐14028*C both from the United Kingdom appear to be on the same fairly extended B haplotype background as each other (B), and interestingly this variant has recently been reported in two other instances (rs759157971), also both in the United Kingdom. The ‐13779*C allele is located on a C haplotype background and is most prevalent in India, where it is clustered geographically (Table S2).

The techniques used in this study provide suggestive evidence of functional significance but may, of course, underestimate effects *in vivo* since the Caco‐2 cell line expresses only low levels of lactase and in some cases shows effects that are not important *in vivo* (Rousset, 1986). The role of these variants could be pursued using cotransection approaches analogous to those on ‐13915 T>G and ‐13910 C>T described previously (Lewinsky et al., 2005; Enattah et al., 2008), to show evidence of the interaction of the transcription factors identified here with other factors involved in lactase expression. However, techniques based on chromatin‐immunoprecipitation such ChIP‐Seq (chromatin immunoprecipitation coupled to high‐throughput sequencing) give a better insight to specific transcription factor DNA targets and their networks in the intestine (e.g. reviewed in Olsen et al., 2012) and could be useful to further examine the influence of the *LCT* enhancer alleles on transcription factor binding or influence of epigenetic modifications on *LCT* expression. Ideally, these experiments should be conducted on isolated intestinal enterocytes, though the chances of obtaining such samples from individuals who carry rare variants are remote. The recent work of Labrie et al. shows that the enhancer region associated with ‐13910*T escapes age‐related DNA methylation (Labrie et al., 2016). It seems likely that changes in transcription factor binding, might be responsible for preventing this methylation, and thus allowing continued adult expression of *LCT* in carriers of this, and the other functional variants. If the *in vitro* effects that we observe represent the *in vivo* situation, one can imagine that the binding of somewhat dynamic complexes of transcription factors is altered by the nucleotide changes and that it is these changes that assist in keeping the chromatin in an open and unmethylated state, thus escaping epigenetic ageing.

This study illustrates the complexities of studying regulatory SNPs (rSNPs) but shows how a combination of experimental and bioinformatic approaches will be needed to identify causal rSNPs in complex disease.

## Supporting information

Disclaimer: Supplementary materials have been peer‐reviewed but not copyedited.

**Table S1‐2, Figures S1‐4**.
