# Supplementary material for: In Vitro Functional Analyses of Infrequent Nucleotide Variants in the Lactase Enhancer Reveal Different Molecular Routes to Increased Lactase Promoter Activity and Lactase Persistence
Source: Ann Hum Genet. 2016 Oct 7;80(6):307–18. doi: 10.1111/ahg.12167 (PMC5129500; doi:10.1111/ahg.12167)
Supplement: Supplementary file 1 — Table S1‐2, Figures S1‐4. [file AHG-80-307-s001.docx]

Supplementary Online data

Supplementary Table 1: Double stranded oligonucleotides used for EMSAs. Variants are indicated in bold.

| **Probe name** | **Sequence** |
| --- | --- |
| **Enhancer variants** |  |
| 14028T (ancestral) | 5'-ACGTCATAGTTTATAGAGTGCATAAA-3' |
|  | 3'-GCAGTATCAAATATCTCACGTATTTC-5' |
| 14028C | 5'-ACGTCATAGTT**C**ATAGAGTGCATAAA-3' |
|  | 3'-GCAGTATCAA**G**TATCTCACGTATTTC-3' |
| Ancestral (14009T, 14010G, 14011C ) | 5'-TAGAGTGCATAAAGACGTAAGTTACCATTTA-3' |
|  | 3'-TCTCACGTATTTCTGCATTCAATGGTAAATT-5' |
| 14011T | 5'-TAGAGTGCATAAAGA**T**GTAAGTTACCATTTA-3' |
|  | 3'-TCTCACGTATTTCT**A**CATTCAATGGTAAATT-5' |
| 14010C | 5'-TAGAGTGCATAAAGAC**C**TAAGTTACCATTTA-3' |
|  | 3'-TCTCACGTATTTCTG**G**ATTCAATGGTAAATT-5' |
| 14009G | 5'-TAGAGTGCATAAAGACG**G**AAGTTACCATTTA-3' |
|  | 3'-TCTCACGTATTTCTGC**C**TTCAATGGTAAATT-5' |
| 13779G (ancestral) | 5'-AGTAGTACGAAAGGGCATTCAA-3' |
|  | 3'-CATCATGCTTTCCCGTAAGTTC-5' |
| 13779C | 5'-AGTAGTAC**C**AAAGGGCATTCAA-3' |
|  | 3'-CATCATG**G**TTTCCCGTAAGTTC-5' |
| **TF binding competitors** |  |
| SIF1 24 (Cdx-2 binding site) | 5'-TGGGTGCAATAAAACTTTATGAGTA-3' |
|  | 3'-CCCACGTTATTTTGAAATACTCATT-5' |
| Oct control | 5'-ATGTCGAATGCAAATCACTAGAA-3' |
|  | 3'-ACAGCTTACGTTTAGTGATCTTA-5' |
| LPH-CE2c  (HNF-1α binding site) | 5'-ATAACCCAGTTAAATATTAAGTCTTAAT-3' |
|  | 3'-TATTGGGTCAATTTATAATTCAGAATTA-5' |
| TREH's HNF4 site  (HNF-4α binding site) | 5'-CCTCAAAGGCTGGACTTTGGCCGACTTGG-3' |
|  | 3'-CTCAAAGGCTGGACTTTGGCCGACTTGGA-5' |
| mut Xbal 13910 site  (2 GATA binding sites) | 5'-TACAGATAAGATAATTCTAGACCTGGCCTCAAAGGA-3' |
|  | 3'-ATGTCTATTCTATTAAGATCTGGACCGGAGTTTCCT-5' |
| unspec24 (non-specific) | 5'-AACGTAGCTGATCGAATCGGTTAC-3' |
|  | 3'-TGCATCGACTAGCTTAGCCAATGA-5' |
| **Newly designed TF binding competitors** |  |
| c-Ets-1 | 5'-AATCCTCTACCGGATGTAGGTCGAC-3' |
|  | 3'-TAGGAGATGGCCTACATCCAGCTGA-5' |
| Ets/Tel-2 | 5'-ATCTTACTACTTCCTCGCTGACCGT-3' |
|  | 3'-AGAATGATGAAGGAGCGACTGGCAA-5' |
| NF-kappaB | 5'-AGTGGCGGGGAAAGTCCCCAGAATC-3' |
|  | 3'-CACCGCCCCTTTCAGGGGTCTTAGA-5' |
| Pax | 5'-AAGAAGTGGAACTCACGATCGTGCT-3' |
|  | 3'-TCTTCACCTTGAGTGCTAGCACGAA-5' |
| Pax4-8 | 5'-ACGGCGTTCATGCGTGAGCGACCGT-3' |
|  | 3'-GCCGCAAGTACGCACTCGCTGGCAA-5' |
| Pbx | 5'-AGATGGGATTGATGGTAGCCGTATT-3' |
|  | 3'-CTACCCTAACTACCATCGGCATAAA-5' |
|  |  |
| **Probe name** | **Sequence** |
| **Competitors from Affymetrix EMSA kit** |  |
| Aff_c-Ets-1 | 5'-ACCAGGAAGCCAGGAAG-3' |
|  | 3'-GGTCCTTCGGTCCTTCA-5' |
| Aff_ETS (1) | 5'-AGGAGGAGGGCTGCTTGAGGAAGTATAAGAAT-3' |
|  | 3'-CCTCCTCCCGACGAACTCCTTCATATTCTTAA-5' |
| Aff_ELF | 5'-AGAGTCATCAGAAGAGGAAAAATGAAGGT-3' |
|  | 3'-CTCAGTAGTCTTCTCCTTTTTACTTCCAA-5' |
| Aff_Elk-1 | 5'-ATTTGCAAAATGCAGGAATTGTTTTCACAGT-3' |
|  | 3'-AAACGTTTTACGTCCTTAACAAAAGTGTCAA-5' |
| Aff_LEF1 | 5'-ACCCATTTCCATGACGTCATGGTTA-3' |
|  | 3'-GGGTAAAGGTACTGCAGTACCAATA-5' |
| Aff_GKLF | 5'-AATGCAGGAGGAGAAAGAAGGGCGTAGTATCTACTAG-3' |
|  | 3'-TACGTCCTCCTCTTTCTTCCCGCATCATAGATGATCA-5' |
| Aff_AML1 | 5'-AATCTCTATGTGGGTGTGGGTGTGGGAATCAT-3' |
|  | 3'-TAGAGATACACCCACACCCACACCCTTAGTAA-5' |
| Aff_GATA3 | 5'-AGTTATTTATCTCTTAGTTGTAGTTATTTATCTCTTAGTTGTA-3' |
|  | 3'-CAATAAATAGAGAATCAACATCAATAAATAGAGAATCAACATA-5' |
| Aff_GATA4 | 5'-AGCCTAAGCCAAGTGATAAGCAGCCAGACAA-3' |
|  | 3'-CGGATTCGGTTCACTATTCGTCGGTCTGTTA-5' |

Supplementary Table 2: Frequencies of the variants *-13779*C* and *-14011*T* in India and in all the groups in which they were found, and all data for those in Tamil Nadu. Full previously published data for Indian populations can be found in Supplemental Table 4 in Gallego Romero *et al*. (2012).

|  |  |  |  |  | ***Allele frequencies*** | | |  | |
| --- | --- | --- | --- | --- | --- | --- | --- | --- | --- |
|  | **Region** | **State** | **District** | **Population** | ***-13779*C*** | ***-14011*T*** | |  | |
| Present study | All India |  |  |  | 0.014 | 0 | |  |  |
| Gallego Romero *et al.,* 2012 | All India |  |  |  | 0.024 | 0.004 | |  |  |
|  | West | Gujarat | Chota Udaipur | Bhil | 0 | 0.017 | |  |  |
|  | West | Gujarat | Rajkot | Lohana | 0.021 | 0.027 | |  |  |
|  | West | Haryana | Karnal | Mixed castes | 0 | 0.036 | |  |  |
|  | *West* | *Haryana* | *Karnal* | Ror | 0 | 0.022 | |  |  |
|  | West | Maharasthra/Madhya Pradesh | Multiple districts | Barela | 0 | 0.033 | |  |  |
|  | West | Rajasthan | Ajmer | Meena | 0.027 | 0 | |  |  |
|  | North | Uttar Pradesh | Multiple districts | Brahmin | 0 | 0.042 | |  |  |
|  | North | Uttar Pradesh | Shahjahanpur | Brahmin | 0 | 0.04 | |  |  |
|  | North | Uttar Pradesh | Mirzapur | Kol | 0 | 0.036 | |  |  |
|  | North | Uttar Pradesh | Jaunpur/Etah | Kshatriya | 0 | 0.036 | |  |  |
|  | North | Uttar Pradesh | Nainital | Tharu | 0.009 | 0 | |  |  |
|  | North | Uttaranchal | Almora | Brahmin | 0 | 0.1 | |  |  |
|  | South | Andhra Pradesh | Adilabad | Naidu | 0.056 | 0 | |  |  |
|  | South | Kerala | Wayanad | Kattunaikkan | 0.146* | 0 | |  |  |
|  |  |  |  |  |  |  |  |  |  |
|  | South | Tamil Nadu | Madurai | Badaga | 0.012 | 0 | |  |  |
|  | South | Tamil Nadu | Madurai | Brahmin | 0.025 | 0 | |  |  |
|  | South | Tamil Nadu | Dindigul | Palyan | 0 | 0 | |  |  |
|  | South | Tamil Nadu | Periakulam | Liggayat | 0 | 0 | |  |  |
|  | South | Tamil Nadu | Madurai | Low caste | 0 | 0 | |  |  |
|  | South | Tamil Nadu | Madurai | Ezhara | 0 | 0 | |  |  |
|  | South | Tamil Nadu | Nilgiri | Irula | 0.042 | 0 | |  |  |
|  | South | Tamil Nadu | Nilgiri | Kota | 0.041 | 0 | |  |  |
|  | South | Tamil Nadu | Nilgiri | Kurumba | 0.123 | 0 | |  |  |
|  | South | Tamil Nadu | Madurai | Saurashtrian | 0.08 | 0 | |  |  |
|  | *South* | *Tamil Nadu* | *Nilgiri* | Toda | 0.091 | 0 | |  |  |
|  | *South* | *Tamil Nadu* | *Madurai* | Yadava | 0.067 | 0 | |  |  |
|  |  |  |  |  |  |  |  | |  |
|  |  |  |  |  |  |  |  | |  |
|  |  | Pastoralists |  |  |  |  |  | |  |
|  |  | Non-pastoralist |  |  |  |  |  | |  |

*Absent from 5 other groups in Kerala

| 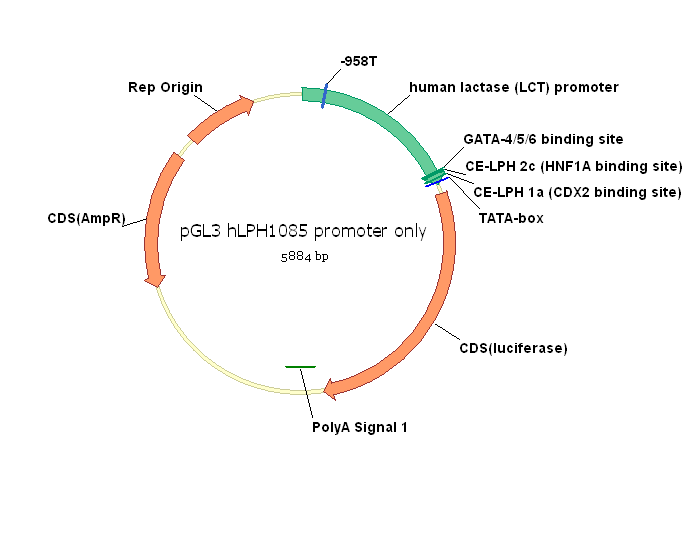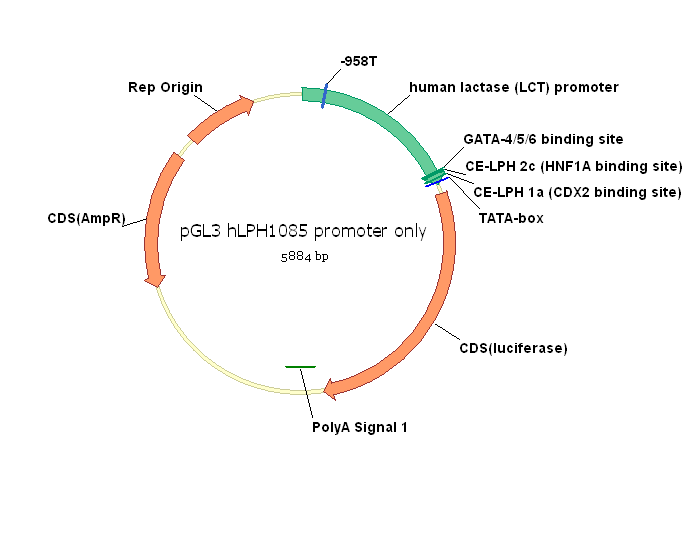 | 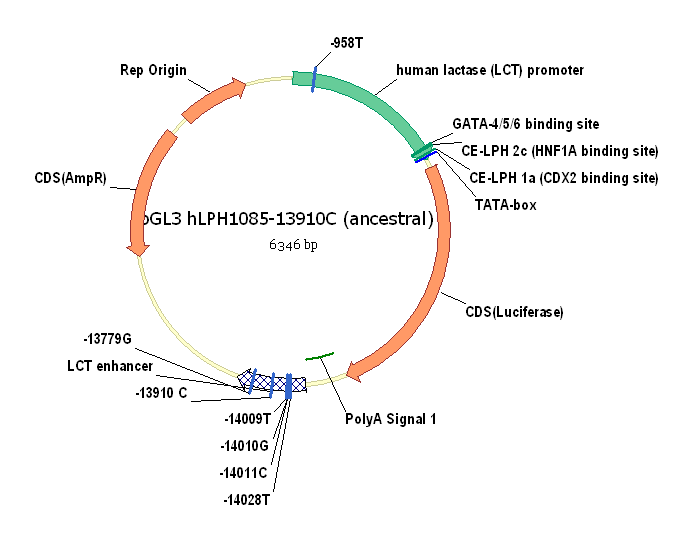 |
| --- | --- |
| 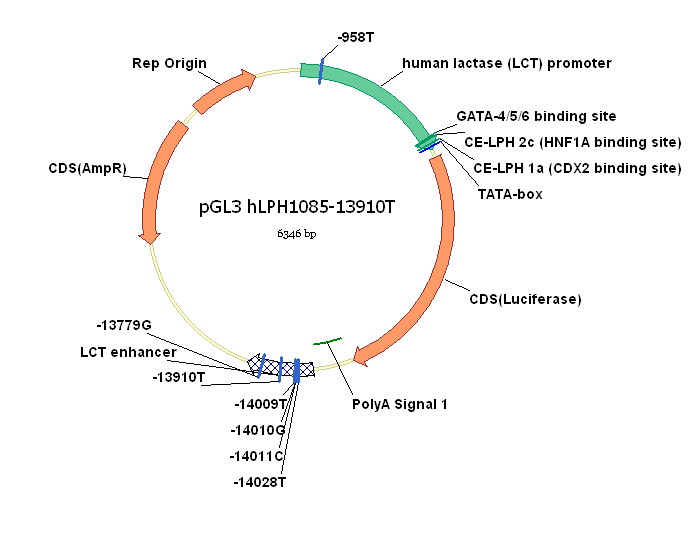 | 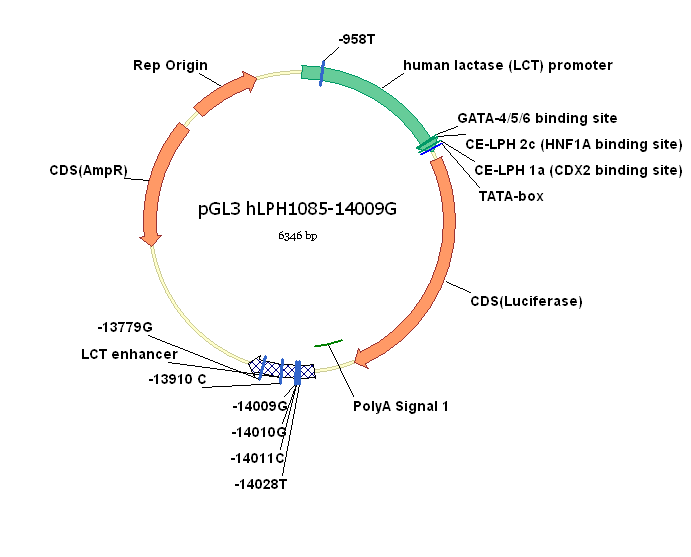 |
| 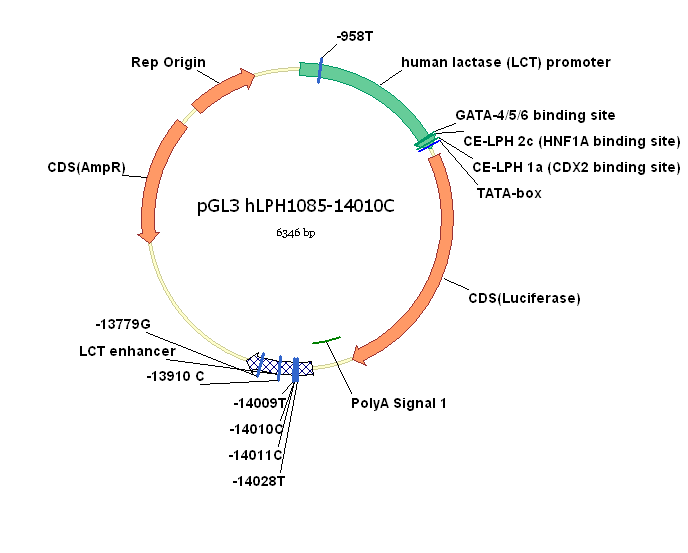 | 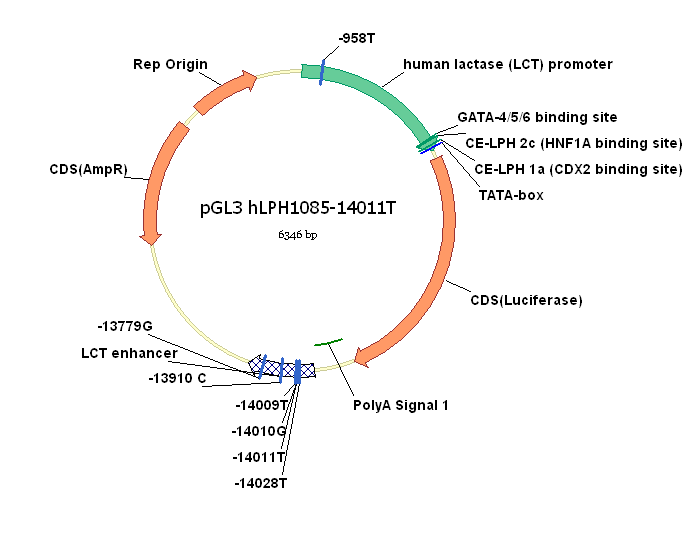 |
| 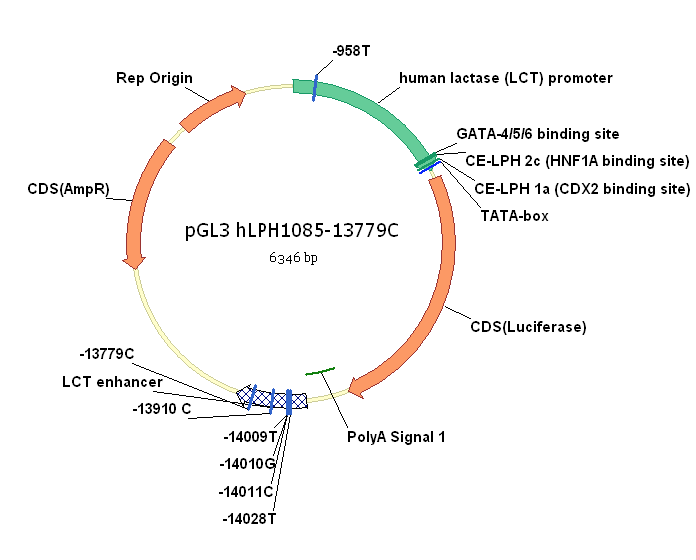 | 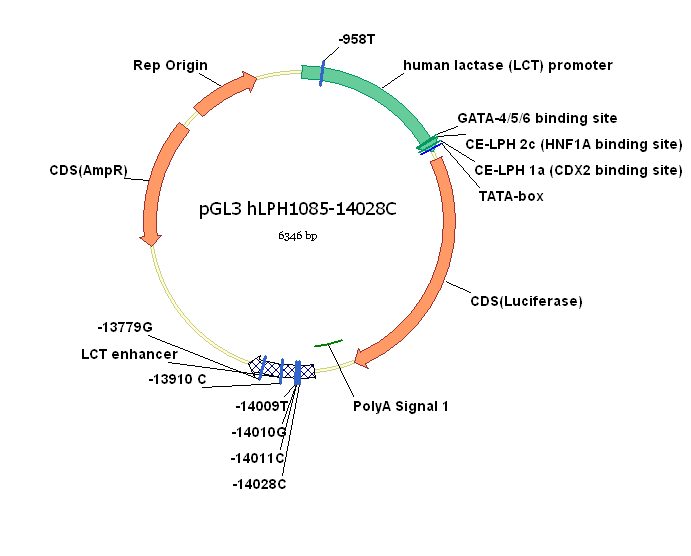 |

**Supplementary Figure 1a: Plasmid maps of the lactase promoter/enhancer constructs.**

ORIGIN

1 cccaggcatt ctgactcctg tataaccatt aagatatgca gagaaagaaa actggaaaga

61 tacatattgc tgaagatact tattatagga agaggagggg ggagggtgaa ggaatttgca

121 agtttttcat agatgtttc**T** atattgtttg aatctcttac aaaatatgtt cagcatattt

181 ttaaagagaa aatttggggc aaaatactta tttttgtatt atgtaaacaa attttaaaat

241 aatgtgtggc tgggtgcgct ggctcacacc tgtaatccca acactttagg aggctgaggc

301 aagaggattg cttgagccca ggagttcaag accagcctgg gtgacatggc aaaactccat

361 ctctactaaa aatacaaaaa attagccagt cgtggtggcg cacacctatg gtcccaccta

421 cccaggatgc tgagatggga ggatcacttg agcccaggaa gtcaaggctg caggaagctg

481 tgatcgcacc actgcactcc cacctgggca acagagtgag acccggtcac caaaaaacaa

541 aaaaaacaaa aaaaattggt aatcgttttc ttcagacatt ttccgggttc ctctgcttaa

601 cttgtatagg aagtctgagg tttttgtgtt ggtctttacc tttttttttt tttttttttt

661 ttttaagatg gagtctcatt ctgttgccca ggctggagtg cagtggcatg atcttggctc

721 ctgcaacctc cgcctcctgg gttcaagtga ttctcctgcc tcagcctcct gagtagccgg

781 gactacaggc gcatgccacg atgcctggct aattttttgt atttttagta gagatggggt

841 ttcaccatgt tagctaggac ggtctcgatc tcctgacctc gtgatccgcc cacctcggcc

901 tcccaaagtg ctggaattac aggtgtgagc caccacgccc ggccctgatc tttacatttt

961 taaatattgc attagtgaac cgtgtactga ttttgtgatc atagataacc cagttaaata

1021 ttaagtctta attatcactt agtattttac aacctcagtt gcagttataa agtaagggtt

1081 ccacatctcg agatctgcga tctaagtaag cttggcattc cggtactgtt ggtaaagcca

1141 ccatggaaga cgccaaaaac ataaagaaag gcccggcgcc attctatccg ctggaagatg

1201 gaaccgctgg agagcaactg cataaggcta tgaagagata cgccctggtt cctggaacaa

1261 ttgcttttac agatgcacat atcgaggtgg acatcactta cgctgagtac ttcgaaatgt

1321 ccgttcggtt ggcagaagct atgaaacgat atgggctgaa tacaaatcac agaatcgtcg

1381 tatgcagtga aaactctctt caattcttta tgccggtgtt gggcgcgtta tttatcggag

1441 ttgcagttgc gcccgcgaac gacatttata atgaacgtga attgctcaac agtatgggca

1501 tttcgcagcc taccgtggtg ttcgtttcca aaaaggggtt gcaaaaaatt ttgaacgtgc

1561 aaaaaaagct cccaatcatc caaaaaatta ttatcatgga ttctaaaacg gattaccagg

1621 gatttcagtc gatgtacacg ttcgtcacat ctcatctacc tcccggtttt aatgaatacg

1681 attttgtgcc agagtccttc gatagggaca agacaattgc actgatcatg aactcctctg

1741 gatctactgg tctgcctaaa ggtgtcgctc tgcctcatag aactgcctgc gtgagattct

1801 cgcatgccag agatcctatt tttggcaatc aaatcattcc ggatactgcg attttaagtg

1861 ttgttccatt ccatcacggt tttggaatgt ttactacact cggatatttg atatgtggat

1921 ttcgagtcgt cttaatgtat agatttgaag aagagctgtt tctgaggagc cttcaggatt

1981 acaagattca aagtgcgctg ctggtgccaa ccctattctc cttcttcgcc aaaagcactc

2041 tgattgacaa atacgattta tctaatttac acgaaattgc ttctggtggc gctcccctct

2101 ctaaggaagt cggggaagcg gttgccaaga ggttccatct gccaggtatc aggcaaggat

2161 atgggctcac tgagactaca tcagctattc tgattacacc cgagggggat gataaaccgg

2221 gcgcggtcgg taaagttgtt ccattttttg aagcgaaggt tgtggatctg gataccggga

2281 aaacgctggg cgttaatcaa agaggcgaac tgtgtgtgag aggtcctatg attatgtccg

2341 gttatgtaaa caatccggaa gcgaccaacg ccttgattga caaggatgga tggctacatt

2401 ctggagacat agcttactgg gacgaagacg aacacttctt catcgttgac cgcctgaagt

2461 ctctgattaa gtacaaaggc tatcaggtgg ctcccgctga attggaatcc atcttgctcc

2521 aacaccccaa catcttcgac gcaggtgtcg caggtcttcc cgacgatgac gccggtgaac

2581 ttcccgccgc cgttgttgtt ttggagcacg gaaagacgat gacggaaaaa gagatcgtgg

2641 attacgtcgc cagtcaagta acaaccgcga aaaagttgcg cggaggagtt gtgtttgtgg

2701 acgaagtacc gaaaggtctt accggaaaac tcgacgcaag aaaaatcaga gagatcctca

2761 taaaggccaa gaagggcgga aagatcgccg tgtaattcta gagtcggggc ggccggccgc

2821 ttcgagcaga catgataaga tacattgatg agtttggaca aaccacaact agaatgcagt

2881 gaaaaaaatg ctttatttgt gaaatttgtg atgctattgc tttatttgta accattataa

2941 gctgcaataa acaagttaac aacaacaatt gcattcattt tatgtttcag gttcaggggg

3001 aggtgtggga ggttttttaa agcaagtaaa acctctacaa atgtggtaaa atcgataagg

3061 atccgtcgag tttatgtaac tgttgaatgc tcatacgacc atggaattct tccctttaaa

3121 gagcttggta agcatttgag tgtagttgtt agacggagac gatcacgtca tagtt**T**atag

3181 agtgcataaa ga**CGT**aagtt accatttaat acctttcatt caggaaaaat gtacttagac

3241 cctacaatgt actagtaggc ctctgcgctg gcaatacaga taagataa**t**g tag**c**cc**c**tgg

3301 cctcaaagga actctcctcc ttaggttgca tttgtataat gtttgatttt tagattgttc

3361 tttgagccct gcattccacg aggataggtc agtgggtatt aacgaggtaa aaggggagta

3421 gtac**G**aaagg gcattcaagc gtcccatctt cgcttcaacc aaagcagccc tgcgttttcc

3481 tagttttatt aataggtttg atgtaaggtc gtctttgaaa agggggctcg accgatgccc

3541 ttgagagcct tcaacccagt cagctccttc cggtgggcgc ggggcatgac tatcgtcgcc

3601 gcacttatga ctgtcttctt tatcatgcaa ctcgtaggac aggtgccggc agcgctcttc

3661 cgcttcctcg ctcactgact cgctgcgctc ggtcgttcgg ctgcggcgag cggtatcagc

3721 tcactcaaag gcggtaatac ggttatccac agaatcaggg gataacgcag gaaagaacat

3781 gtgagcaaaa ggccagcaaa aggccaggaa ccgtaaaaag gccgcgttgc tggcgttttt

3841 ccataggctc cgcccccctg acgagcatca caaaaatcga cgctcaagtc agaggtggcg

3901 aaacccgaca ggactataaa gataccaggc gtttccccct ggaagctccc tcgtgcgctc

3961 tcctgttccg accctgccgc ttaccggata cctgtccgcc tttctccctt cgggaagcgt

4021 ggcgctttct catagctcac gctgtaggta tctcagttcg gtgtaggtcg ttcgctccaa

4081 gctgggctgt gtgcacgaac cccccgttca gcccgaccgc tgcgccttat ccggtaacta

4141 tcgtcttgag tccaacccgg taagacacga cttatcgcca ctggcagcag ccactggtaa

4201 caggattagc agagcgaggt atgtaggcgg tgctacagag ttcttgaagt ggtggcctaa

4261 ctacggctac actagaagaa cagtatttgg tatctgcgct ctgctgaagc cagttacctt

4321 cggaaaaaga gttggtagct cttgatccgg caaacaaacc accgctggta gcggtggttt

4381 ttttgtttgc aagcagcaga ttacgcgcag aaaaaaagga tctcaagaag atcctttgat

4441 cttttctacg gggtctgacg ctcagtggaa cgaaaactca cgttaaggga ttttggtcat

4501 gagattatca aaaaggatct tcacctagat ccttttaaat taaaaatgaa gttttaaatc

4561 aatctaaagt atatatgagt aaacttggtc tgacagttac caatgcttaa tcagtgaggc

4621 acctatctca gcgatctgtc tatttcgttc atccatagtt gcctgactcc ccgtcgtgta

4681 gataactacg atacgggagg gcttaccatc tggccccagt gctgcaatga taccgcgaga

4741 cccacgctca ccggctccag atttatcagc aataaaccag ccagccggaa gggccgagcg

4801 cagaagtggt cctgcaactt tatccgcctc catccagtct attaattgtt gccgggaagc

4861 tagagtaagt agttcgccag ttaatagttt gcgcaacgtt gttgccattg ctacaggcat

4921 cgtggtgtca cgctcgtcgt ttggtatggc ttcattcagc tccggttccc aacgatcaag

4981 gcgagttaca tgatccccca tgttgtgcaa aaaagcggtt agctccttcg gtcctccgat

5041 cgttgtcaga agtaagttgg ccgcagtgtt atcactcatg gttatggcag cactgcataa

5101 ttctcttact gtcatgccat ccgtaagatg cttttctgtg actggtgagt actcaaccaa

5161 gtcattctga gaatagtgta tgcggcgacc gagttgctct tgcccggcgt caatacggga

5221 taataccgcg ccacatagca gaactttaaa agtgctcatc attggaaaac gttcttcggg

5281 gcgaaaactc tcaaggatct taccgctgtt gagatccagt tcgatgtaac ccactcgtgc

5341 acccaactga tcttcagcat cttttacttt caccagcgtt tctgggtgag caaaaacagg

5401 aaggcaaaat gccgcaaaaa agggaataag ggcgacacgg aaatgttgaa tactcatact

5461 cttccttttt caatattatt gaagcattta tcagggttat tgtctcatga gcggatacat

5521 atttgaatgt atttagaaaa ataaacaaat aggggttccg cgcacatttc cccgaaaagt

5581 gccacctgac gcgccctgta gcggcgcatt aagcgcggcg ggtgtggtgg ttacgcgcag

5641 cgtgaccgct acacttgcca gcgccctagc gcccgctcct ttcgctttct tcccttcctt

5701 tctcgccacg ttcgccggct ttccccgtca agctctaaat cgggggctcc ctttagggtt

5761 ccgatttagt gctttacggc acctcgaccc caaaaaactt gattagggtg atggttcacg

5821 tagtgggcca tcgccctgat agacggtttt tcgccctttg acgttggagt ccacgttctt

5881 taatagtgga ctcttgttcc aaactggaac aacactcaac cctatctcgg tctattcttt

5941 tgatttataa gggattttgc cgatttcggc ctattggtta aaaaatgagc tgatttaaca

6001 aaaatttaac gcgaatttta acaaaatatt aacgcttaca atttgccatt cgccattcag

6061 gctgcgcaac tgttgggaag ggcgatcggt gcgggcctct tcgctattac gccagcccaa

6121 gctaccatga taagtaagta atattaaggt acgggaggta cttggagcgg ccgcaataaa

6181 atatctttat tttcattaca tctgtgtgtt ggttttttgt gtgaatcgat agtactaaca

6241 tacgctctcc atcaaaacaa aacgaaacaa aacaaactag caaaataggc tgtccccagt

6301 gcaagtgcag gtgccagaac atttctctat cgataggtac cgagct

**Supplementary Figure 1b: Sequence of the pGL hLPH1085-13910C vector construct with the region immediately upstream of *LCT* (highlighted in yellow) and the ancestral sequence of the enhancer (highlighted in green).** Positions of the variant enhancer alleles featuring in this paper and the -958 position are shown in capital letters in red bold. Positions of the previously studied functional variants at -13907, -13910 and -13915 are shown in lower case.


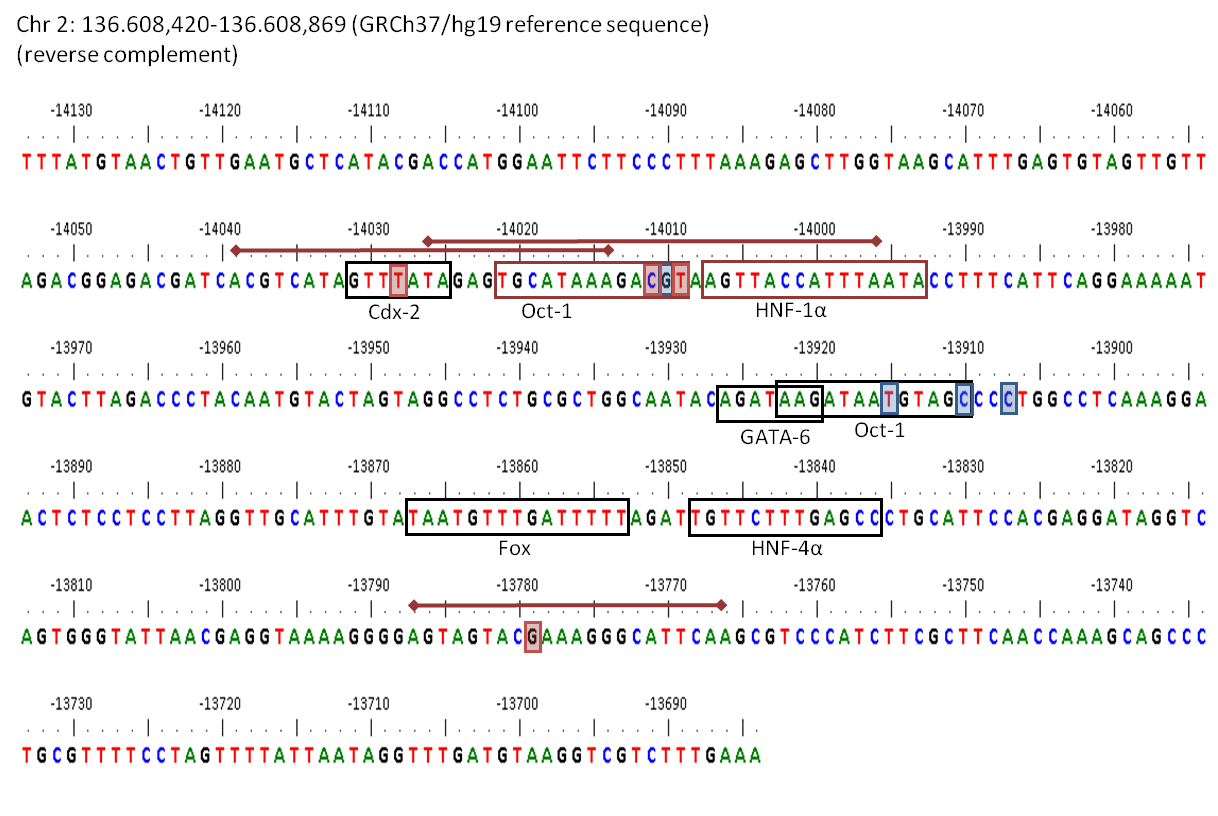


Supplementary Figure 2: Positions in the 450bp *LCT* enhancer of transcription factor binding sites and SNPs studied functionally. Indicated in boxes: transcription factor binding sites as previously shown in experiments before, black: Lewinsky *et al*. (2005) and red: Jensen *et al.* (2011), blue shadowed: SNPs previously examined functionally (Enattah *et al*., 2008, Ingram *et al*., 2007, Olds *et al*., 2011, Tishkoff *et al*., 2007); red shadowed: SNPs examined functionally in this study. EMSA probes used in this study are indicated with red lines


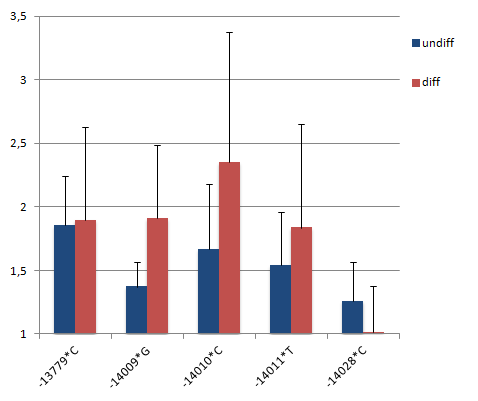


Supplementary Figure 3: Summary of all experiments showing luciferase reporter gene assays for all *LCT* enhancer variants tested in undifferentiated (undiff) and differentiated (diff) Caco-2 cells (after 2 and 9 days of transfection respectively). Luciferase activity of enhancer variant constructs are shown in fold activity compared to the ancestral variant construct. Luciferase activities (means, n=16 in 4 independent experiments for undifferentiated and 20 in 4 separate experiments for differentiated cells) were corrected for transfection efficiency against ß-galactosidase and normalized to the expression of pGL3 hLPH1085 (promoter only). Consistently increased promoter expression was shown for all *except* the *-14028*C* variant and was statistically significant (p<0.05) as tested with a two way ANOVA following Bonferroni correction for multiple testing.


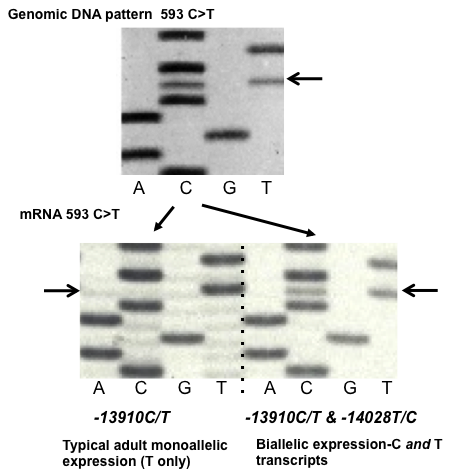


**Supplementary Figure 4: Homozygous high (biallelic) expression of lactase mRNA in a -13910 C/T heterozygote who is also heterozygous for -14028 T/C, detected using a SNP in *LCT,* exon 1, 593 C>T.** The first gel fragment shows genomic DNA from a 593 C>T heterozygote. The second shows the same piece of sequence in duodenal mRNA converted to cDNA from two 593 C>T heterozygotes, both heterozygous for -13910 C/T, but the second person also heterozygous for -14028 T/C. PCR products subjected to Sanger sequencing as described in (Wang *et al*., 1998) (pre-capilliary era sequencing, in which the sequence can be read from the bottom to the top of the gel).

The first mRNA sample shows a typical -13910 C/T, 593 C/T adult, where only the T allele of heterozygote is detectable in the RNA, while the second sample from the person who is also heterozygous for -14028 T/C shows equal high expression of the C and T carrying transcripts. Similar results were obtained with 666 G>A (rs3754698), 5579 T>C (rs2278544). The combined data revealed that the B haplotype transcript, as well as the usual A haplotype, is highly expressed in this person. Figure constructed using data taken from Hollox (2000), Poulter *et al*. (2003) and Wang *et al*. (1995).
